# Distal oblique bundle influence on distal radioulnar joint stability: a biomechanical study

**DOI:** 10.1038/s41598-023-48875-y

**Published:** 2023-12-07

**Authors:** G. Hohenberger, F. Pirrung, N. Hammer, J. A. Niestrawska

**Affiliations:** 1 Department of Trauma Surgery, State Hospital Feldbach-Fürstenfeld, Feldbach, Austria; 2 Division of Macroscopic and Clinical Anatomy, Gottfried Schatz Research Center, Medical University of Graz, Graz, Austria; 3 Department of Orthopedic and Trauma Surgery, University of Leipzig, Leipzig, Germany; 4 Division of Biomechatronics, Fraunhofer Institute for Machine Tools and Forming Technology, Dresden, Germany

**Keywords:** Ligaments, Implants, Characterization and analytical techniques, Biological physics

## Abstract

Chronic instability of the distal radioulnar joint (DRUJ) presents a highly disabling condition. Several surgical techniques have been reported for its treatment. These involve reconstruction of the distal oblique bundle (DOB) of the interosseous membrane (IOM) of the forearm. The aim of this study was to examine whether surgical reconstruction of the DOB is necessary to restore DRUJ stability following trauma with DOB disruption and to compare two restoration techniques utilizing a tendon or suture-button graft. Stability in supination and pronation was assessed by means of maximum torque and force in twenty forearms. Test cycles were performed with the DOB/IOM in an intact condition, with the DOB or distal IOM transected, and following surgical reconstruction of the DOB with either tendon graft or suture-button system. In pronation, the relative change in maximum axial force was significantly lower in samples with a transected DOB in comparison to samples without a preexisting DOB. No statistically significant differences were observed between forearms including DOB reconstruction and specimens in the intact and transected state. Neither were there statistically significant differences concerning the two surgical techniques. From a biomechanical perspective, surgical DOB reconstruction is hence not indicated in cases of isolated DOB rupture.

## Introduction

Instability of the distal radioulnar joint (DRUJ) represents a commonly missed diagnosis and is often associated with Galeazzi fractures of the distal forearm^1^. Further causes of DRUJ instability include lesions to the triangular fibrocartilage complex (TFCC) and Essex-Lopresti injuries^2^. It also forms a concomitant injury of fractures of the distal radius in up to 35% of cases^3–5^. Chronic DRUJ instability results in a highly disabling condition, including ulnar-sided wrist pain, grip strength weakness, painful and limited pronation/supination as well as degenerative arthritis as long-term sequalae^4–7^. Several surgical procedures have been postulated for reinforcing the soft tissue stabilizers of the DRUJ. These involve capsular plication, an advancement of pronator quadratus^8^, reinserting the triangular fibrocartilage complex^9^, reconstruction of the distal radioulnar ligaments (Adams–Berger technique)^10,11^, and the reconstruction of the distal oblique bundle (DOB) of the interosseous membrane (IOM) of the forearm. The latter structure is considered an enhancement of the most distal portion of the IOM, which collectively act as a secondary stabilizer of the DRUJ^12^. Surgical stabilization of the DOB may provide a surgical alternative to the reconstruction technique by Adams and Berger^13^ and has been topic of interest of recent clinical studies involving smaller cohorts^14,15^. Brink and Hannemann^6^ and Verbeek et al.^16^ reported satisfactory outcomes concerning reconstruction of the DOB using tendinous grafts. In contrast, this stabilization procedure may also be performed using suture-button constructs. Both tendon^17–19^ and suture-button graft^20^ techniques have previously been evaluated biomechanically. These previous studies^17–20^ assessed the volar/dorsal and/or total translation of the distal radius in relation to the ulna. Additionally, the isolated DOB reconstruction had been evaluated^20^ or compared to the Adams and Berger technique^17–19^. Therefore, the literature lacks studies assessing the stability along the physiological rotation axis of the forearm and comparison between tendon graft and suture-button systems.

The aim of this given study was to examine the biomechanical stability of the DRUJ in situ under a physiological loading scenario in supination and pronation within three possible conditions: (1) with or without a pre-existing DOB in an uninjured state, (2) with the DOB or distal IOM transected, and (3) following surgical DOB reconstruction utilizing a tendon or suture-button graft. For an isolated assessment of the role of the DOB, the respective TFCC and distal radioulnar ligaments were left in an intact state.

It was hypothesized that the DOB acts as a main stabilizer of the DRUJ reflected by the maximum force drop in pronation/supination following its transection, requiring surgical stabilization in cases of DOB lesions so to prevent instability.

## Materials and methods

### Specimens

The sample included 21 upper extremities from eleven human corpses (four females, seven males) which had been embalmed using a modified composition of the Thiel method^21^. The age at death of the collective averaged 80.5 years (SD 12.2 years; range 57–95 years). The respective individuals, while alive, gave their written and informed consent to donate their bodies for research and teaching to the Division of Macroscopic and Clinical Anatomy of the Medical University of Graz. The study was approved by the ethics committee of the Medical University of Graz (approval number: 34-533 ex 21/22). All methods were carried out in accordance with relevant guidelines and regulations. One upper extremity was excluded from the experiments owing an ulna fracture during the preparation process. The resulting collective involved ten left and ten right, eighteen paired and two unpaired arms. None of these remaining specimens showed signs of preceding interventions or trauma in the area of interest and all specimens exhibited intact TFCC and distal radioulnar ligaments.

### Preparation

Prior to the biomechanical experiments, the forearm and elbow regions were removed from the humerus approximately 12 cm proximal to the humeral epicondyles. The distal portion of the hand was detached at the level of the intercarpal line. All specimens were removed from soft tissues with the exception of the TFCC, ligaments, joint capsules and the IOM. For each forearm, the extensor indicis tendon was harvested as a graft for surgical reconstruction. Concerning the aims of this study, each ten forearms with and without a DOB were included. A 4.0-mm Schanz screw (DePuy Synthes, Oberdorf, Switzerland) was inserted horizontally into the distal portion of the radius for the mounting in the biomechanical testing device, 10 mm proximal to the watershed line.

### Mechanical testing

**Figure 1:**
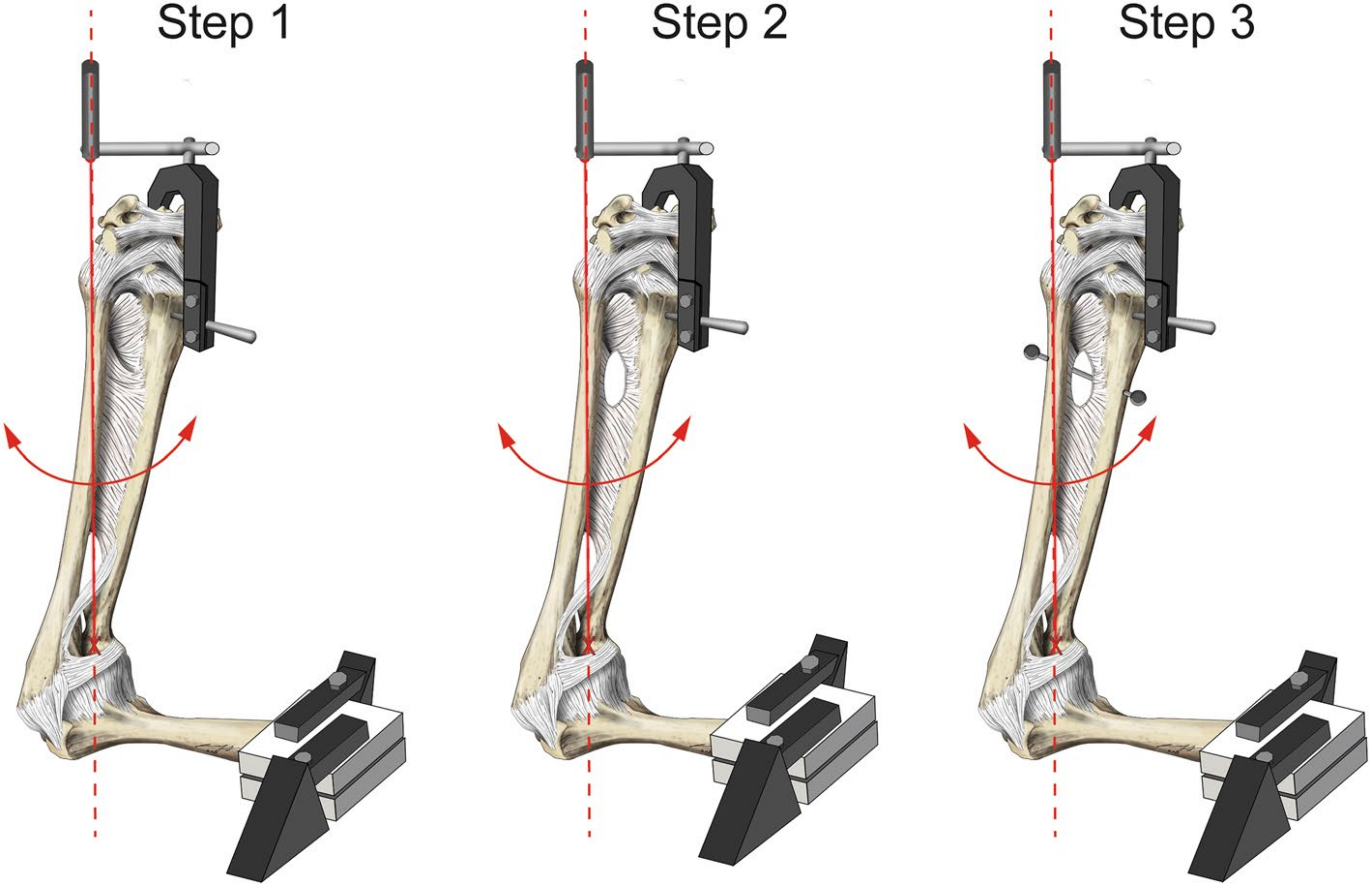
Schematic depiction of the steps before each experimental cycle. Step 1 shows a prepared specimen without a preexisting DOB. Step 2 shows the IOM transected distally, and step 3 depicts the condition following surgical repair. The red line denotes the rotational axis which was determined by a laser gauge, adjusting the ulnar styloid process and the head of the radius positioned onto the same axis. The arrows indicated the directions of rotation.

Following the dissection procedure, each specimen was mounted in the Z020 torsion multi-axis testing system, equipped with a Serie M torque cell (nominal torque 200 Nm) and a Xforce HP load cell to measure axial forces (nominal force 500 N; all ZwickRoell AG, Ulm, Germany). The mechanical testing set up is illustrated in Fig. 1. A 4.0 Schanz Screw was fixed between the axis of rotation of the distal radioulnar joint and the axis of rotation of the machine, utilizing a custom-made 3D-printed apparatus. The specimen was adjusted so that the ulnar styloid process and the head of the radius positioned in the same axis^22^. A laser gauge was used to determine accurately the rotational axis. The humerus was then mounted with a 3D-printed chuck jaw to be positioned horizontally, keeping the rotational axis in place.

Interventions and measurements were performed with the following steps, further outlined below.

#### Step 1

As the starting point of the mechanical trial the neutral position of the forearm was chosen, denoting a 0°-rotation. Supination and pronation movements were chosen to examine the stabilizing effect of the IOM or the DOB, executed until a torsion angle of 100° was reached, which is larger than an anticipated maximum active range of motion, 85° for supination and 90° for pronation under physiological conditions^22^. To avoid viscoelastic effects, the axis was then rotated quasi-statically. Each loading step was performed three times and the axial force and momentum resulting from the induced rotation were measured at the third step to account for any preconditioning effects.

#### Step 2

As a next step, all of the specimens were prepared for simulated DOB failure, or in cases of an absent DOB the failure of the distal portion of the IOM. The according incisions were performed with a scalpel with the specimens mounted in the testing apparatus. If a DOB was present, its insertion sites were marked to ease the (later) placement of a repair graft before the DOB was incised and removed. In cases of nonexistence of the DOB, the distal portion of the IOM was incised longitudinally, starting from the DRUJ to the level of the proximal border of pronator quadratus. This level was chosen due to the coincidence of the origin of the proximal insertion of the DOB and the proximal border of the pronator quadratus^23^. Following the incision, the testing protocol was repeated as stated above, rotating quasi-statically in the range of 100°-0°-100° three times.

#### Step 3

**Figure 2:**
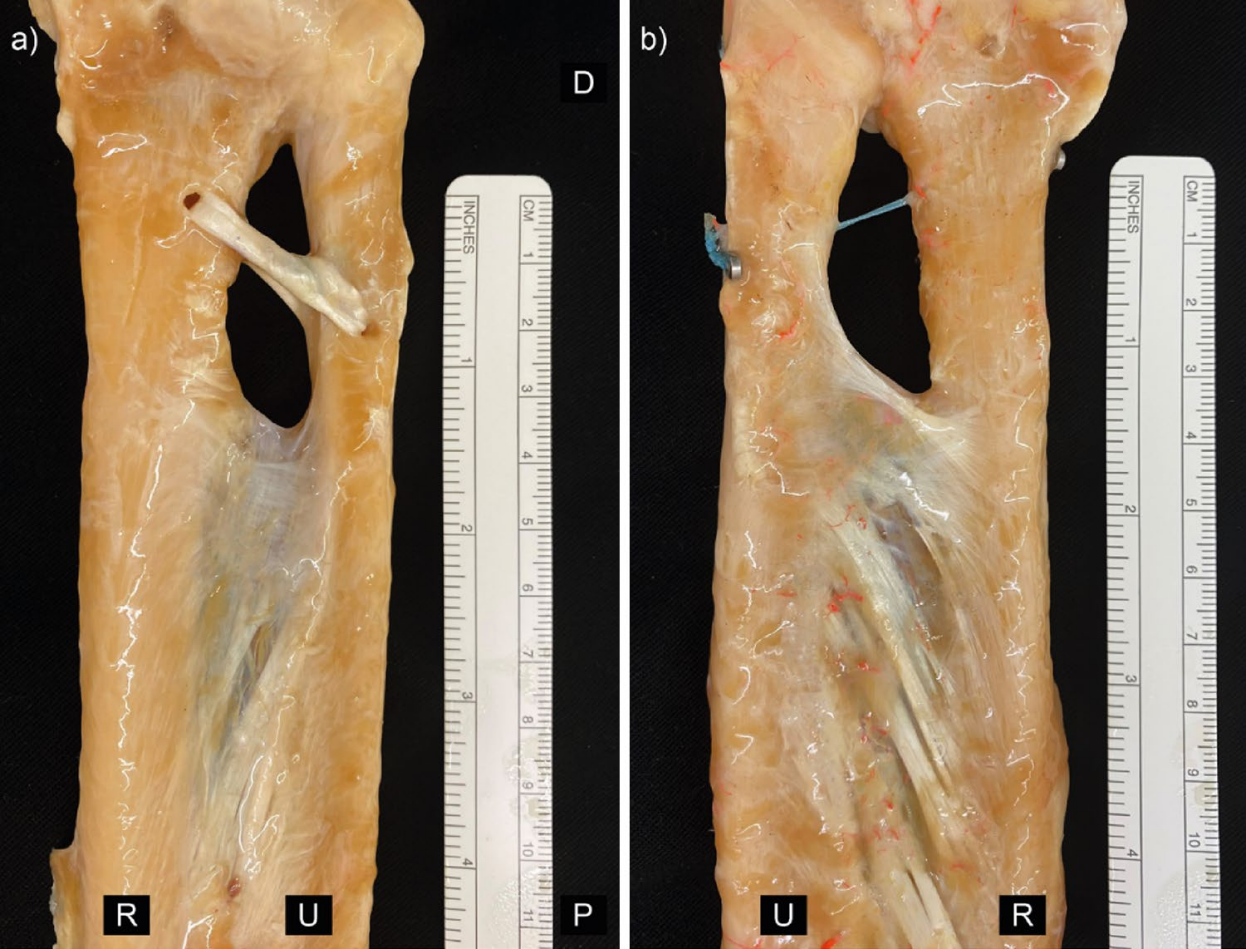
Photographs of (**a**) the volar aspect of a left forearm following DOB reconstruction with extensor indicis tendon graft (**b**) volar depiction of a right forearm including DOB reconstruction with the suture-button system. *U* ulnar, *R* radial, *P* proximal, *D* distal. The background has been changed to black.

Following the second step of the experiments, five specimens including and five arms without a DOB underwent reconstruction of the DOB by use of an extensor indicis tendon graft (see Figs. 1 and 2a, Step 3). The technique for the surgical reconstruction was performed as described by Riggenbach and colleagues^13^. If no DOB was present, the insertion levels were chosen as reported by Hohenberger et al.^24^, 30 mm proximal to the tip of the radial styloid process and 42 mm proximal to the ulnar styloid process. Care was taken to maintain a 3- to 5-mm bone bridge above the drill holes. The ends of the tendon graft were narrowed to ease the fitting through the tunnels and the graft subsequently pulled through the drill holes of the radius and the ulna using a suture passer (Arthrex GmbH, Munich, Germany). The graft ends were fixed via a Pulvertaft suture including stabilization using 2.0 nonabsorbable suture material (FiberWire®; Arthrex GmbH, Munich, Germany). When the suture was performed, the forearm had to be brought to 90° of supination to maximally tension the graft.

Additionally, besides the tendon reconstruction technique, five forearms with a DOB and five without received surgical stabilization of the DOB via a suture-button system (TightRope® Attachable-Button-System; Arthrex GmbH) (see Fig. 2b). The drill holes were positioned obliquely through the ulna and radius at the heights described above. Following insertion of the ThightRope® and two buttons, the construct was secured with at least five knots, which were tightened with the forearm in 90° supination. Both reconstruction techniques are shown in Fig. 2.

**Figure 3:**
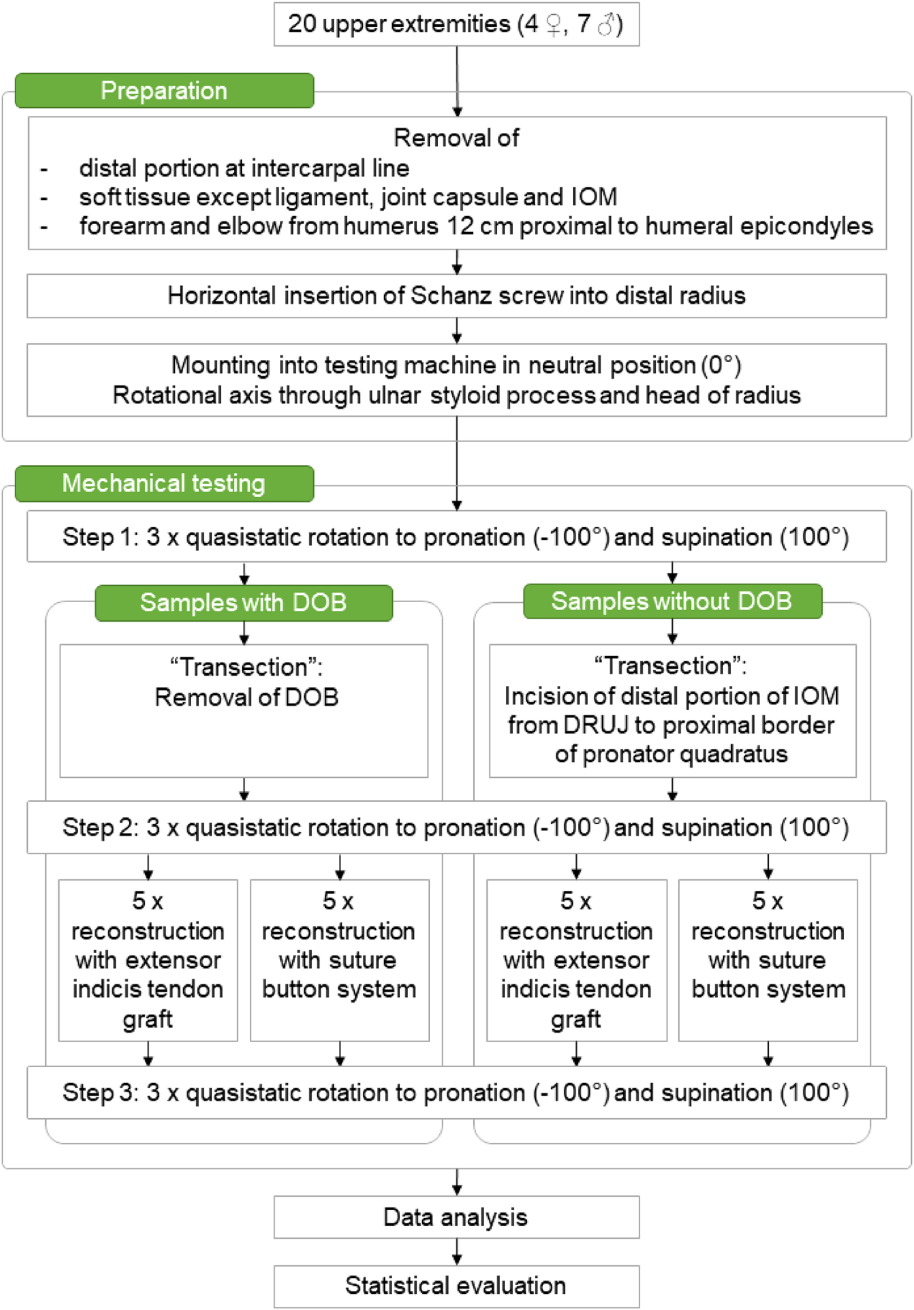
Flowchart depicting the workflow from sample preparation to statistical evaluation. *IOM* interosseous membrane, *DRUI* distal radioulnar joint, *DOB* distal oblique bundle.

All specimens were then again tested as stated above. For a schematic depiction of the workflow see Fig. 3.

### Processing of mechanical data

The maximum torque M_max,sup_ at supination and M_max,pro_ [Nm] at pronation as well as the maximum forces F_max, sup_ at supination and F_max, pro_ [N] at pronation were evaluated from the third measuring cycle of each specimen and state, respectively. To account for inter-individual and side-dependent difference in bone geometry, the relative change between the three interventions was evaluated instead of comparing absolute values. For illustrative purposes, the relative change of F_max, pro_ before and after transection of the DOB was calculated as follows: Relative change = F_max, pro_ of transected state/F_max, pro_ of the control state. This yielded values < 1 if F_max,pro_ of the transected state was higher than F_max, pro_ of the control state, or values > 1 if F_max, pro_ of the transected state was lower than F_max, pro_ of the control state. A value of 1 hence denotes no change relative to the compared group.

### Statistical analyses

Statistical evaluation was performed using GraphPad Prism (v. 9.4.1, GraphPad Software, LLC, LaJolla, CA, USA). Significant differences between the individual groups were tested by means of the one-way analysis of variance (ANOVA) and a Bonferroni correction. Normal distribution was assessed with means of the Shapiro–Wilk test. Differences were considered statistically significant at *p* values ≤ 0.05. Data are reported as mean values and standard deviation (mean ± STD).

## Results

**Figure 4:**
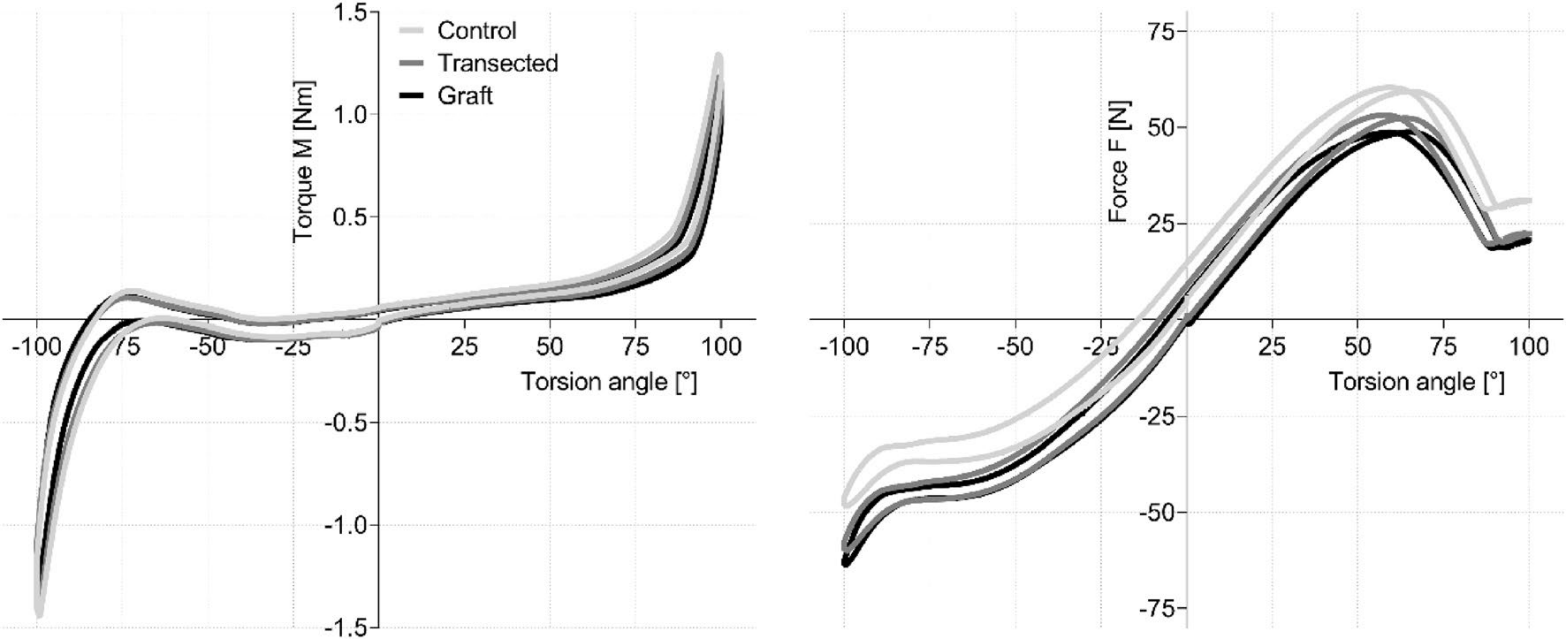
Torque–angle (left) and force-angle (right) graphs for an exemplary sample, showing three different curves each for the intact condition (step 1, light grey), the transected condition (step 2, darker grey) and the repaired condition using a “graft” (step 3, black).

Comparison between the intact IOM/DOB and the transected state yielded no difference in torque nor axial force, in neither pronation nor supination, respectively. Graphs for an exemplary sample showing measured torque M and axial force F are depicted in Fig. 4.

No statistically significant changes were observed between tendon or graft repaired samples in either of the groups when compared to the control group nor when compared to the transected group in terms of maximum torque in pronation M_max,pro_ nor in supination M_max,sup_. Similarly, no significant difference was observed when comparing the two repair techniques with each other.

No statistical difference was found for any of the measurements between left- and right-sided specimens.

The relative change in maximum force in pronation F_max,pro_ between the cut samples in comparison to the uncut control samples were significantly different between specimen with versus with no DOB present (DOB 0.88 ± 0.11, without DOB 1.00 ± 0.14, p = 0.02).

**Figure 5:**
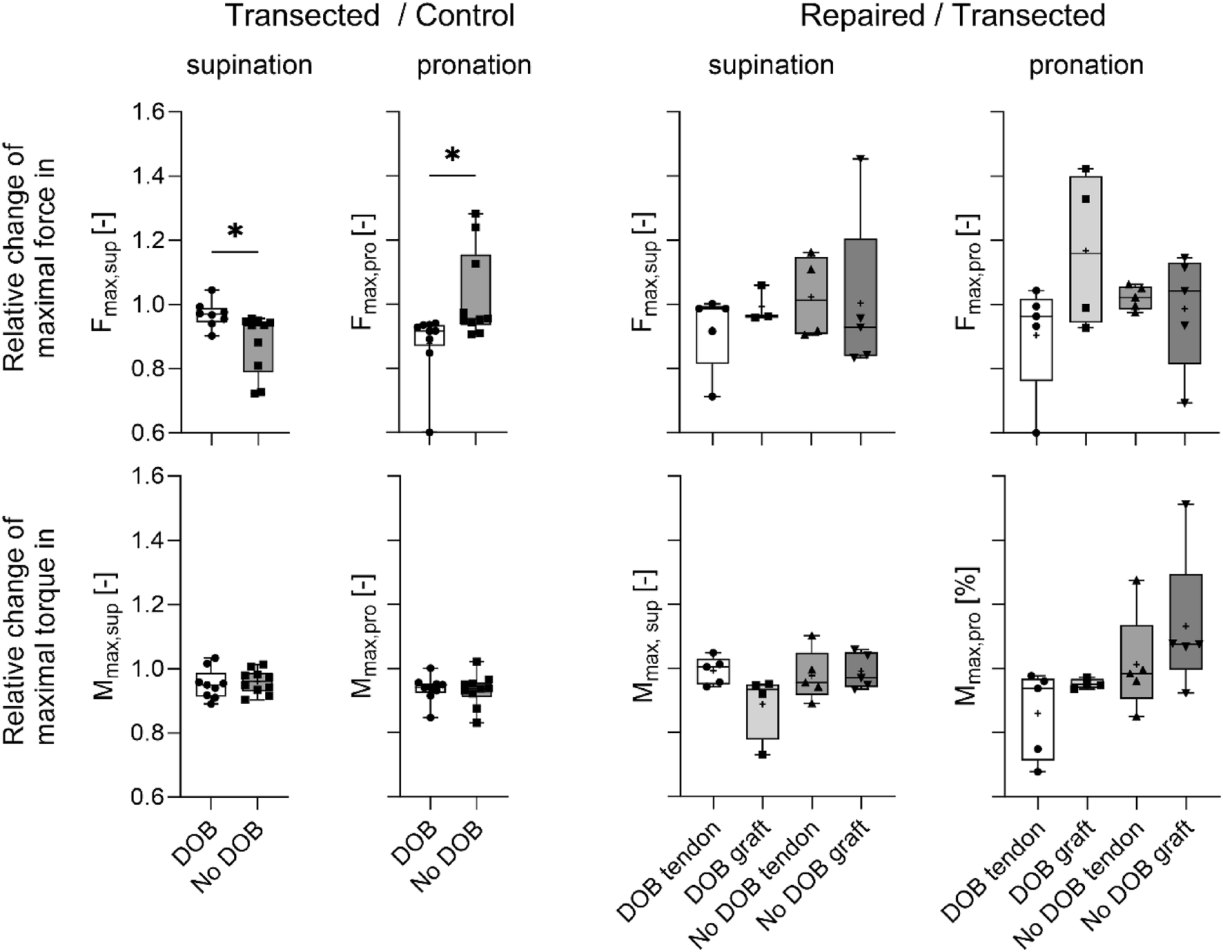
Box-and-whisker-plots of relative change in maximum force and torque in pronation and supination for transected vs. control and repaired vs. transected specimen. Control samples include specimens without a DOB (‘No DOB’) and specimens with DOB. *DOB* distal oblique bundle. *p < 0.05, box-and-whisker plots of median, maximum and minimum values and 25–75 percentile. + depicts the mean value.

The relative change of force in supination F_max,sup_ was significantly different between cut and uncut control samples as well (DOB 0.97 ± 0.04, without DOB 0.88 ± 0.09). For box-and-whisker-plots of all comparisons between transected vs. control and repaired vs. transected samples see Fig. 5.

When comparing the relative changes between the repaired and transected samples no significant changes could be seen. Tables 1 and 2 summarize all relative changes between the examined groups.

**Table 1:** Values of the change from cut to control samples with and without a DOB in measured force and torque.

	Transected/control F_max,pro_		Transected/control F_max,sup_	
	DOB	No DOB	DOB	No DOB
Mean	0.88	1.00	0.97	0.88
STD	0.11	0.14	0.04	0.09

	Transected/control M_max,pro_		Transected/control M_max,sup_	
	DOB	No DOB	DOB	No DOB
Mean	0.94	0.93	0.95	0.96
STD	0.04	0.05	0.05	0.04

*DOB* distal oblique bundle, *F*_*max,pro*_ maximum force at pronation, *F*_*max,sup*_ Maximum force at supination, *M*_*max,pro*_ maximum torque at pronation, *M*_*max,sup*_ Maximum torque at supination.

**Table 2:** Changes induced by repair in contrast to the transected samples are summarized in this table.

	DOB tendon	DOB graft	No DOB tendon	No DOB graft
Repaired/transected F_max,pro_				
Mean	0.90	1.20	1.00	0.99
STD	0.18	0.25	0.04	0.18
Repaired/transected F_max,sup_				
Mean	0.92	0.99	1.00	1.00
STD	0.12	0.06	0.13	0.26
Repaired/transected M_max,pro_				
Mean	0.86	0.95	1.00	1.11
STD	0.14	0.02	0.16	0.22
Repaired/transected M_max,sup_				
Mean	0.99	0.89	0.98	0.99
STD	0.043	0.11	0.08	0.06

Two groups of samples have been tested here: samples with a DOB present and without a DOB. They have either been repaired with a tendon or with a graft after being transected in the second step. Values of the change of graft versus cut samples with and without a DOB, with tendon or graft repair, respectively. *DOB* distal oblique bundle, *F*_*max,pro*_ maximum force at pronation, *F*_*max,sup*_ Maximum force at supination, *M*_*max,pro*_ maximum torque at pronation, *M*_*max,sup*_ Maximum torque at supination.

## Discussion

The aim of this study was to evaluate if surgical stabilization of the DOB following trauma was essential, and to assess potential differences between two surgical reconstruction techniques (tendon graft versus suture-button system) for this anatomical characteristic. Since we aimed to exclusively assess the meaningfulness of DOB stabilization, the respective TFCC and distal radioulnar ligaments of the utilized specimens were left in an intact state.

### Maximum force in pronation decreases following DOB transection but remains unaffected in samples without a DOB following transection of the distal IOM

Maximum axial force in pronation F_max,pro_ was significantly lower in samples with transected DOB when compared to the intact condition following transection for samples with a DOB. No such difference was observed in samples without a DOB present in the intact state. Here, maximum axial force in pronation remained similar following transection of the distal part of the IOM. Other than that, we did not observe any significant biomechanical difference, neither in pronation nor supination regarding the incision of the distal IOM or the DOB. Lee et al.^25^ in their retrospective magnetic resonance imaging and clinical evaluation study on DRUJ stability found decreased rates of joint instability in patients with TFCC lesions who had a DOB when compared to individuals without this IOM enhancement. This may be corroborated by our findings, as we observed a decreased maximum axial force in pronation following transection of the DOB when compared to the intact condition in the subgroup with a preexisting DOB. This was not applicable for the specimens without a DOB. However, we did not transect the TFCC in our sample.

### Surgical reconstruction yields no difference when compared to the transected state.

Surgical reconstruction of the transected DOB yielded no difference in stability under pronation nor supination when compared to the control or transected state.

The stabilizers of the DRUJ involve pronator quadratus, the TFCC, its osseous configuration and the articular capsule^26^. Among these structures, the deep ligamentous portions of the TFCC represent the main intrinsic stabilizers of the joint^16,27–29^. However, the distal portion of the IOM, including the DOB, has been described to serve as a secondary stabilizer of the DRUJ^23–27,30,31^, especially when the TFCC is torn^12, 32, 33^, e.g., in the setting of distal radius fractures^5^. The DOB has been reported at an incidence ranging between 20 up to 87.5%^17–19,34–38^. Its most distal fibers blend into the volar and dorsal radioulnar ligaments of the TFCC^24,39,40^.

Kitamura and colleagues^27^ observed that the subgroup with an intact DOB yielded significantly increased DRUJ stability in a neutral position when compared to the group without DOB. These results corroborate the findings by Werner et al.^41^. Dy and colleagues^42^ reported that a coronal shift of 2 mm of a distal radius fracture combined with fracture of the ulnar styloid process may lead to increased DRUJ displacement in specimens with a DOB, when compared to those without this structure. The few available clinical studies concerning DOB reconstruction via tendinous graft in patients with chronic DRUJ instability have also reported satisfactory postsurgical outcomes^6,16^. However, the latter two studies solely report reconstruction of the DOB including uninjured TFCC. The present results indicate that surgical reconstruction of the DOB is unnecessary when the TFCC in untorn and the DOB’s contribution as a stabilizer of the DRUJ is negligible. Concerning biomechanical trials involving DOB reconstruction, previous studies mainly assessed the dorsal/volar translation of the distal portion of the radius with reference to the ulnar head. Riggenbach and colleagues^19^ found that in pronation, complete DOB repairs were significantly more stable when compared to the partial and incomplete states. Reconstructions did further not differ significantly from the intact DRUJ. In neutral position and supination, the reconstructions improved instability, however without statistical significance. Delbast et al.^17^ compared the stability of the intact DRUJ to an unstable condition following transection of the distal IOM and the TFCC and following DOB stabilization by Riggenbach et al.^13^. They found a major instability in all specimens following transection of the TFCC and the distal IOM. Even following DOB reconstruction, major instability remained present in 25% of all DRUJs at 45° supination.

The current study yielded no statistically significant differences concerning DRUJ stability between specimens following DOB reconstruction with an intact DOB/distal IOM in pronation nor supination. Our methods further focused on the forearm subjected to physiological loading during supination and pronation in vitro, reflecting the condition in vivo, since although performed by for example Riggenbach et al.^19^, no translation of the radius against the ulna is possible under physiological loading conditions due to the stabilization via the local soft tissues. De Vries et al.^20^ evaluated the dorsal translation of the distal radius relative to the ulna in intact specimen, an unstable state (transected volar and dorsal radioulnar ligaments, articular disc and distal IOM) and following suture-button reconstruction of the DOB. Radial translation increased significantly following transection of the stabilizers of the DRUJ when compared to the intact state. We assessed a statistically significant decrease of the maximum axial force during pronation in specimens following transection of the DOB when compared to the intact specimens (solely in the sample including preexisting DOB). Furthermore, the aforementioned trials differed from the here given study concerning the artificially constructed DRUJ instability, since these involved transection of the TFCC, whereas in our sample, solely the distal portion of the IOM was incised. Considering the function of the TFCC to serve as the main primary stabilizer of the DRUJ^27–29^, the varying differences to the here given results may be traced back to the fact that this complex was left intact to focus on the influence of the DOB itself.

## Conclusion

It was found that under physiological loading conditions, the DOB does not seem to have a main stabilizing effect on the DRUJ. Concerning these results, surgical reconstruction of the DOB, if present, appears unnecessary from a biomechanical perspective when the TFCC is intact. A decrease in maximum axial force during pronation was found following transection of the DOB. None of the repair techniques yielded a significant change in stability when compared to the transected state. There were no significant differences between the tendon graft and the suture-button system reconstruction techniques.

## Limitations

This study is limited by the number of available samples and the advanced age (mean: 80.5 years) of the donors. The samples were chemically embalmed, which may impact the mechanical properties to a certain yet unknown extent. However, all samples were treated similarly and the project focused on relative changes to help address these limitations.

## Data Availability

The datasets generated during and/or analyzed during the current study will be made available from the corresponding author upon reasonable request.
